# Structural Stability and Performance of Noble Metal-Free SnO_2_-Based Gas Sensors

**DOI:** 10.3390/bios2020221

**Published:** 2012-05-29

**Authors:** Antonio Tricoli

**Affiliations:** Department of Mechanical and Process Engineering, ETH Zurich, CH-8092 Zurich, Switzerland; E-Mail: atricoli@ethz.ch; Tel.: +41-44-632-8503; Fax: +41-44-632-1276

**Keywords:** gas sensors, SnO_2_, semiconductors, chemoresistive, nanoparticles, long-term stability, grain growth, relative humidity, noble metals, SiO_2_

## Abstract

The structural stability of pure SnO_2_ nanoparticles and highly sensitive SnO_2_-SiO_2_ nanocomposites (0–15 SiO_2_ wt%) has been investigated for conditions relevant to their utilization as chemoresistive gas sensors. Thermal stabilization by SiO_2_ co-synthesis has been investigated at up to 600 °C determining regimes of crystal size stability as a function of SiO_2_-content. For operation up to 400 °C, thermally stable crystal sizes of *ca*. 24 and 11 nm were identified for SnO_2_ nanoparticles and 1.4 wt% SnO_2_-SiO_2_ nanocomposites, respectively. The effect of crystal growth during operation (T_O_ = 320 °C) on the sensor response to ethanol has been reported, revealing possible long-term destabilization mechanisms. In particular, crystal growth and sintering-neck formation were discussed with respect to their potential to change the sensor response and calibration. Furthermore, the effect of SiO_2_ cosynthesis on the cross-sensitivity to humidity of these noble metal-free SnO_2_-based gas sensors was assessed.

## 1. Introduction

Metal-oxide nanoparticles of a few nanometers such as SnO_2_ are excellent materials for the fabrication of solid-state gas sensors [1]. Highly porous film morphologies constituted by such nanoparticles have remarkable potential for several novel and demanding applications such as non-invasive medical diagnostics [2]. However, small nanoparticles suffer from poor thermal stability resulting in insufficient long-term stability of the sensing properties (e.g., baseline and calibration drifts) already at standard operation temperatures (250–500 °C) [3]. Utilization of large particles allow synthesis of thermally more stable film structures with, however, considerably lower sensitivity [4]. In fact, the sensitivity of SnO_2_ nanoparticles increases drastically with decreasing grain size toward twice their Debye length (*ca*. 6 nm at 300 °C) [4,5]. Furthermore, small variation of the neck size between large particles is possible during operation and may also result in a drift of the sensing properties.

Often, addition of noble metals (Ag and Pt) is utilized to enhance the sensitivity of large SnO_2_ grains by the spill-over effect [6]. However, clustering and growth of the noble metal on the metal-oxide surface during sensor operation is in itself a potential long-term destabilization mechanism [3]. Additionally, deposition of noble metals on the metal-oxide surface increases material cost and process complexity. Noble metal-free enhancement of the sensitivity of SnO_2_ nanoparticles has been achieved, recently, by flame-cosynthesis of SiO_2_ [7]. Optimized SnO_2_-SiO_2_ nanocomposites have demonstrated both drastically higher sensor response and short-term thermal stability [7]. 

The long-term stability of these nanocomposites and the effect of SiO_2_ cosynthesis on other important sensors properties such as the cross-sensitivity to humidity have not been investigated so far [7,8,9]. Deposition of flame-made SiO_2_ led to the synthesis of super-hydrophilic surfaces suggesting that its cosynthesis may increase the cross-sensitivity of SnO_2_-based gas sensors to humidity [10,11]. Furthermore, flame-synthesis of SiO_2_ at low precursor concentration has shown that SiO_2_ does not nucleate as easily as other oxides (e.g., SnO_2_ and TiO_2_) [9,11] and thus may condense directly on the surface of the SnO_2_ nanoparticles. Surface localized SiO_2_ could act as active center for the adsorption of H_2_O molecules. This would limit the utilization of SnO_2_-SiO_2_ nanocomposites to constant humidity applications and may even result in a drastic drop of their sensing performance [9]. 

The long-term stability of nanoparticles is dependent on their grain size and sintering/operation temperature [3,12] Although, SiO_2_ cosynthesis has demonstrated to drastically decrease sintering rates [7], the thermodynamically stable crystal and grain sizes of SnO_2_-SiO_2_ nanocomposites have not been determined. In particular, films made of fine nanoparticles may require a very long time to reach sufficient structural stability [12] causing a continuous drift of their electronic properties [3]. The thermal stabilization mechanism of SiO_2_ also plays an important role in the resulting sensor performance dynamics and requires further investigation [7,13,14]. 

Here, we investigate the long-term structural stability of flame-made SnO_2_-SiO_2_ nanocomposites focusing on the electronic properties of the resulting sensing films. Temperature-dependent thermally stable sizes are identified for pure SnO_2_ nanoparticles and tailored SnO_2_-SiO_2_ nanocomposites. The effect of crystal growth during sensor operation is investigated and discussed with respect to possible long-term instability mechanisms. Furthermore, the effect of SiO_2_ cosynthesis on the cross-sensitivity to humidity of the resulting gas sensors is critically compared to that of pure SnO_2_.

## 2. Experimental Section

Pure SnO_2_ nanoparticles with 12 and 21 ± 0.5 nm average crystal size (d_XRD_) and tailored SnO_2_-SiO_2_nanocomposites were produced by flame spray pyrolysis (FSP) as previously described [7,15]. In particular, the deposition time was 4 min for all sensors and was followed by a 30 s *in situ* annealing step at 14 cm HAB with a particle-free xylene flame (12 mL/min) leading to a film bulk thickness of *ca*. 0.8 μm. It is expected that SiO_2_ formation may slightly increase the visible film thickness due to its lower density and by inhibiting SnO_2_ grain sintering. During deposition the substrate back temperature was 120–130 °C as measured by a n-type thermocouple. Furthermore, nanoparticles were collected on water cooled glass-fiber filters placed at 50 cm height above the burner (HAB) and characterized by transmission electron microscopy (TEM) with a Hitachi H600 microscope, operated at 100 kV. X-ray diffraction (XRD) patterns were obtained by a Bruker, AXS D8 Advance diffractometer operated at 40 kV, 40 mA at 2θ (Cu Kα) = 15–75°, step = 0.03 and scan speed = 0.6°/min. The d_XRD_ was determined using the Rietveld fundamental parameter method with the structural parameters of cassiterite [16]. Sintering studies were performed by placing the nanoparticles in a furnace (Carbolite) in air under atmospheric pressure. 

The nanoparticle specific surface area (SSA) was measured by BET analysis using a Micromeritics Tristar 3000. The BET equivalent diameter (d_BET_) was calculated using the density of SnO_2_ and SiO_2_. Sensing films were obtained by direct impingement of the FSP aerosol and *in situ* flame annealing on alumina substrates with Au interdigitated electrodes as previously described [7,15]. The sensor measurements were performed as described in detail elsewhere [17]. The analyte mixture was EtOH (105 ppm ± 3% synthetic air, Pan Gas 5.0) in synthetic air (20.8% ± 2% O_2_ rest nitrogen, Pan Gas 5.0). Water vapor was supplied by an air flow let through a bubbler kept at 20 °C. The temperature was measured with a n-type thermocouple [17]. The sensor response (S) was defined as in Equation (1) [18]:


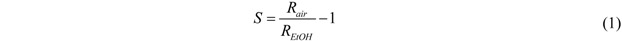


where R_air_ is the film resistance in air with a given relative humidity (r.h.) and R_EtOH_ is the film resistance with a given concentration of ethanol at the same r.h.. The cross-sensitivity (CS) to humidity was defined as [9]: 


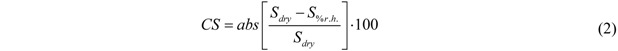


where S_dry_ is the response in dry air and S_%r.h._ is the response at a given r.h. as defined in Equation (1).

## 3. Results and Discussion

### 3.1. Long-Term Structural Stability of SnO_2_-SiO_2_ Nanocomposites

**Figure 1:**
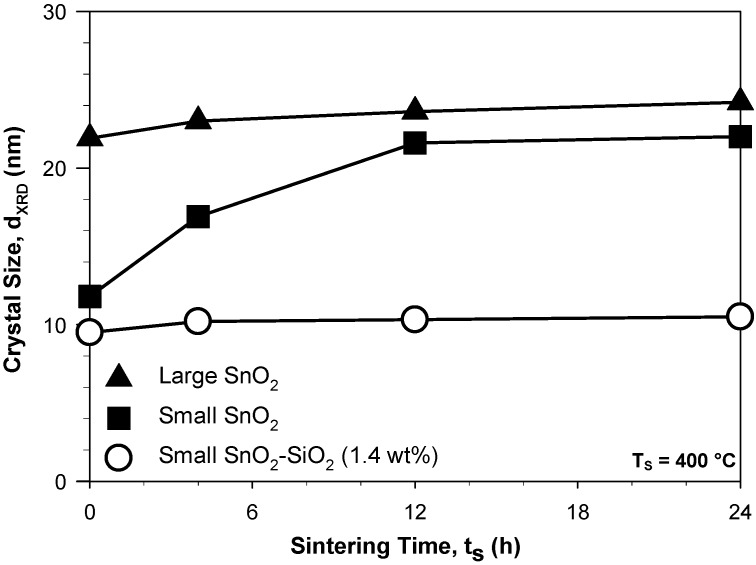
Average crystal size (d_XRD_) of large (triangles) and small (squares) SnO_2_ nanoparticles as a function of the sintering time at 400 °C. Cosynthesis of 1.4 wt% SiO_2_ (circles) drastically increased the long-term thermal-stability of SnO_2_ crystals.

Crystal growth during operation at the elevated working-temperatures (250–500 °C) of metal-oxide gas sensors is considered to contribute to the drift of their baseline (film resistance without the analyte) and poor long-term stability of their response [2,3]. The BET and XRD size were within 1 nm suggesting formation of mainly monocrystalline particles. Figure 1 shows the average crystal size of large (triangles) and small (squares) SnO_2_ nanoparticles as a function of the sintering time at 400 °C. The crystal size of the better performing, small SnO_2_ nanoparticles (Figure 1, squares) increased from 12 to 22 nm with increasing sintering time from 0 to 24 h. A crystal size of 21.6 nm was obtained already after 12 h sintering (Figure 1, squares). In contrast, the crystal size of the large SnO_2_ nanoparticles (Figure 1, triangles) increased only from 21.9 to 24.2 with increasing sintering time from 0 to 24 h. This indicates that, for sensor operation at 400 °C, flame-made nanoparticles constituted by pure SnO_2_ crystals have a thermodynamically stable size of nearly 24 nm in agreement with the poor thermal stability of small SnO_2_ nanoparticles and with the grain size stability conditions reported for several other synthesis methods [3]. This is in line with the rapid crystal and grain growth of flame-made SnO_2_ nanoparticles observed already at low sintering temperatures [7] suggesting that obtaining stable sensor responses requires testing of the sensors for several consecutive days. In particular, the asymptotic-like growth of the small SnO_2_ nanoparticles (Figure 1, squares) toward 22 nm suggests that small drift of sensor response and baseline may continue for a very long time span (>>24 h). In fact, the thermodynamically stable crystal size (at 400 °C) of 24 nm was still not obtained upon 24 h sintering.

**Figure 2:**
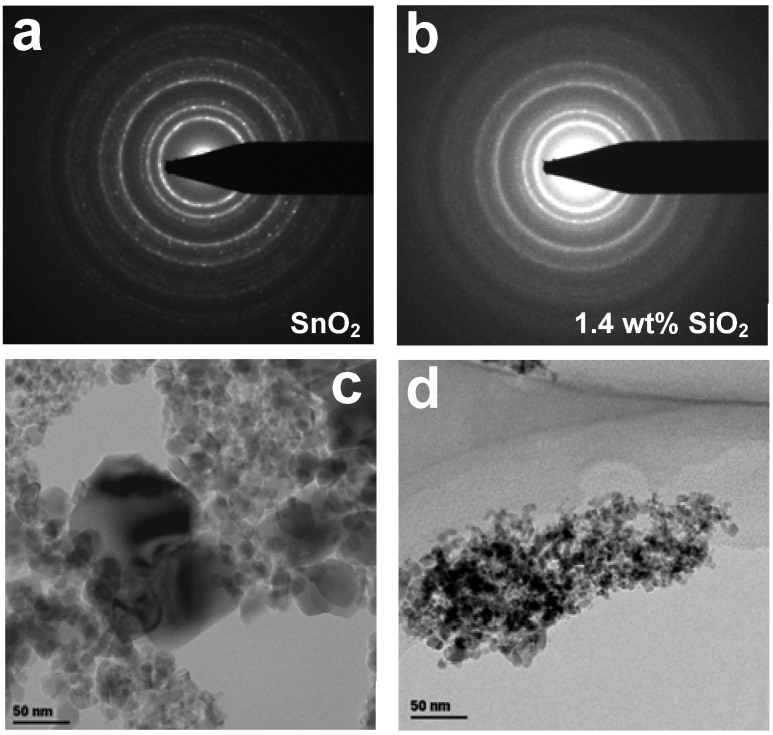
ED patterns of pure (**a**) SnO_2_ nanoparticles and (**b**) 1.4 wt% SnO_2_-SiO_2 _nanocomposites after 4 h sintering at 400 °C and corresponding transmission electron microscopy (TEM) images (**c,d**).

Cosynthesis of SiO_2_ led already at very low content (1.4 wt%) to remarkable long-term thermal-stabilization of the small SnO_2_ nanoparticles. The average crystal size of these SnO_2_-SiO_2_ nanocomposites (Figure 1, circles) increased only from 9.5 to 10.5 nm with increasing sintering time from 0 to 24 h. The particles were mainly polyhedrical (Figure 2(c,d)) consisting of a crystalline SnO_2_ core and some dispersed SiO_2_ phase. At high SiO_2_ content, the SnO_2_ and SiO_2_ phase were segregated in crystalline and amorphous domains, respectively. This is in line with the reported thermal stabilization and performance maximization of SnO_2_- [7] and WO_3_-based [19] gas sensors by Si-doping. Here, it is shown that this noble metal-free approach to improve the performance of metal-oxide chemoresistive gas sensors offers also superior long-term structural stability. 

The thermal stabilization mechanism of the small SnO_2_ crystals by SiO_2_ cosynthesis was further investigated by electron diffraction analysis, XRD and TEM analysis of the nanoparticles upon sintering for 4 h at 400 °C. In line with previous results [7], increasing the SiO_2_ content increased the homogeneity of the visible SnO_2_ TEM size both for the as-prepared and sintered samples. The electron diffraction (ED) patterns of the small SnO_2_ nanoparticles (Figure 2(a)) showed the presence of crystalline structures in line with the XRD analysis [7] that corresponded to 100 wt% cassiterite phase [16] while the numerous bright spots indicate the formation of large crystals already upon short (4 h) sintering at 400 °C. This is in line with the rapid increase in average crystal size of the small SnO_2_ nanoparticles (Figure 1, squares) with increasing sintering time and suggests a polydisperse crystal size distribution. In contrast, the ED patterns of the 1.4 wt% SnO_2_-SiO_2_ nanocomposites (Figure 2(b)) did not shown nearly any bright spot. This indicates that the formation of large crystals is homogeneously inhibited by cosynthesis of SiO_2_. In this respect, a possible thermal stabilization mechanism is the pinning of the SnO_2_ crystal boundaries by SiO_2_ [13,14]. Condensation of the SiO_2_ molecules on the surface of the nucleated SnO_2_ clusters during flame-synthesis may explain the lower sintering rates of these SnO_2_-SiO_2_ nanoparticles already at very low SiO_2_ content. In fact, it is expected that SiO_2_ segregates from the SnO_2_ already at low content (*ca*. 2 wt%) [7].

This homogeneous inhibition of SnO_2_ crystal growth is necessary to achieve long-term stability of the sensing properties. In fact, irregular growth of some un-stabilized SnO_2_ nanoparticles would also lead to change in the structural and electronic properties of the sensing film. However, the crystal size of the SnO_2_-SiO_2_ nanocomposites (Figure 1, circles) was not completely stable and approached slowly 10.5 nm. Although, this crystal growth is very small with respect to that of pure SnO_2_ (Figure 1, squares), it still indicates a restructuring of the nanoparticle interface. In particular, growth of sintering necks may drastically change the performance of the sensing film while showing very small variations in the measured crystal size [7]. 

**Figure 3:**
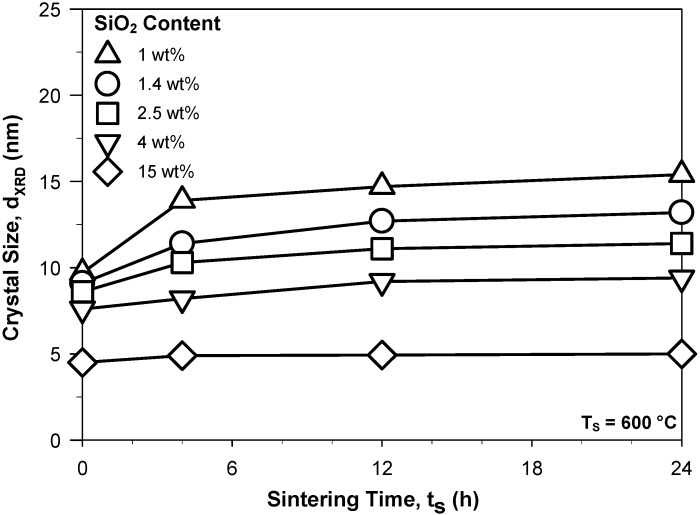
Average SnO_2_ crystal size (d_XRD_) of the SnO_2_-SiO_2_ nanocomposites as a function of the sintering time at 600 °C for several SiO_2_ contents.

Reduction of the long-term drift of the SnO_2_ and SnO_2_-SiO_2_ sensors may be obtained by pre-sintering of the films at temperatures above the operational ones (e.g., at 600 °C) [7] leading to the achievement of a thermodynamically stable grain size prior to sensor testing (e.g., at 400 °C). Figure 3 shows the average SnO_2_ crystal size of several SnO_2_-SiO_2_ nanocomposites as a function of such a pre-sintering step at 600 °C. The 1 wt% SnO_2_-SiO_2_ crystal size (Figure 3, triangles up) increased from 9.7 to 15.4 with increasing sintering time at 600 °C from 0 to 24 h. This shows that even the smallest addition of SiO_2_ leads to stabilization of the SnO_2_ crystal size far below that of pure SnO_2_ at 400 °C (Figure 1, solid triangles). In particular, the 1.4 wt% SnO_2_-SiO_2_ reached a size of 11.4 nm already after 4 h sintering (Figure 3, circles). This is more than the thermodynamically stable size (≈10.5 nm) at 400 °C and thus pre-sintering of the sensing films prior to sensor utilization may be utilized to considerably shorten the time required for achievement of a stable sensor response. Furthermore, up to 4 wt% SiO_2_, the as-prepared SnO_2_ crystal size (Figure 3) of these nanocomposites was very close (*ca*. 10 ± 1.5 nm) suggesting further that SiO_2_ may condense directly on the formed SnO_2_ nanoparticles inhibiting further crystal and grain growth during flame-synthesis. In comparison, the initial crystal size of the pure SnO_2_ nanoparticles was 12 nm (Figure 1, solid squares) which is attributed to particle coagulation during the residence time in the flame. In line, the as-prepared powder SSA increased from 100 to 211 m^2^/g with increasing SiO_2_ content from 0 to 15 wt%. The 15 wt% SiO_2_-SnO_2_ demonstrated the highest long-term stability growing only from 4.5 to 5 nm (Figure 3, diamonds) with increasing sintering time from 0 to 24 h. This is in agreement with the grain growth inhibition demonstrated by SiO_2_ cosynthesis [7]. However, utilization of such high SiO_2_ contents results in the formation of insulating domains and a drastic drop of the sensing performance [7] and thus, here, the dynamics of the sensor response stabilization has been investigated at low SiO_2_ content (1–4 wt%).

### 3.2. Sensing Performance Stability

**Figure 4:**
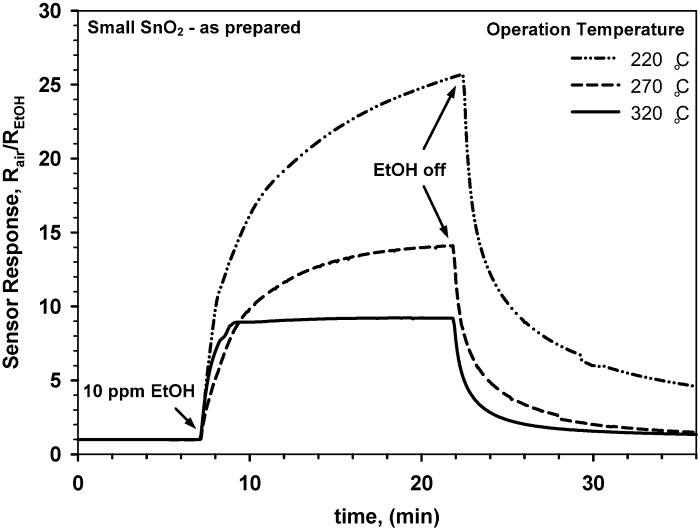
Response of a gas sensor made of as prepared SnO_2_ nanoparticles (d_XRD_ = 12 nm) to 10 ppm ethanol as a function of time for increasing operation temperature from 220 to 320 °C.

**Figure 5:**
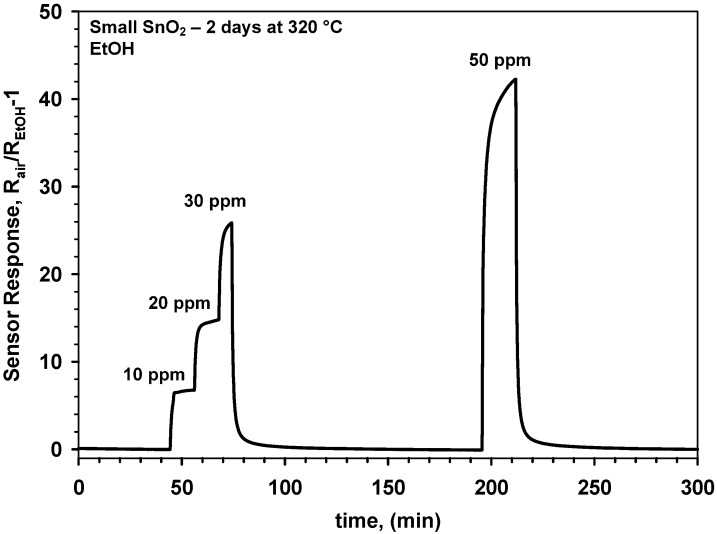
Response of a gas sensor made of small SnO_2_ nanocrystals to ethanol without prior stabilization upon two days at 320 °C in dry air.

The sensing properties of these SnO_2_-SiO_2_ nanoparticles were tested with EtOH, a standard volatile organic compound that is particularly important for detection of drunken drivers and is increasingly investigated also for non-invasive breath analysis. Figure 4 shows the response to 10 ppm ethanol of a pure SnO_2_ (d_XRD_ = 12 nm) gas sensor, that was not stabilized by a pre-sintering step, for several operation temperatures. The response of this sensor (Figure 4) decreased considerably with increasing operation temperature from 220 to 320 °C. This is surprising as pure SnO_2_ has maximal response to EtOH at around 300–350 °C [20]. The drop in the sensor response was attributed to the sintering of the SnO_2_ nanoparticles already during operation at such moderate temperatures. This is in line with the measured crystal growth of the small SnO_2_ nanoparticles (Figure 1, solid squares) that is expected to drastically reduce their sensitivity [3,5,7]. Additionally, operation of the SnO_2_ sensor at 220 °C was characterized (Figure 4, dotted line) by an unstable response and it was not possible to fully recover the initial baseline. This indicates that without prior stabilization the sensing behavior of pure SnO_2_ nanoparticles is characterized by very poor long-term stability. After two days at 320 °C (Figure 4, solid line), the sensor properties were considerably more stable demonstrating a well-defined response to 10 ppm EtOH and full recovery of the initial baseline. Nevertheless, increasing the EtOH concentration to 30 and 50 ppm (Figure 5) resulted in very long response times. 

The step-wise increase of the EtOH concentration (Figure 5) from 10 to 30 ppm at 320 °C demonstrated sufficient sensor sensitivity for discerning among small (<10 ppm) EtOH variations. Furthermore, a very good recovery of the initial baseline was observed (Figure 5) in line with the single EtOH step at 320 °C (Figure 4, solid line). However, the sensitivity of this SnO_2_ sensor to EtOH was pretty low with respect to noble-metals [6] or metal-oxide [7] doped nanoparticles. This was also attributed to the growth of the SnO_2_ crystals during operation and that was found to undermine the reproducibility of the sensor performance. To have more stable sensing properties, novel sensors made of pure SnO_2_ nanoparticles (d_XRD_ = 12 nm) were also tested for EtOH sensing after a sintering step at 600 °C (12 h). Their performance was compared to the sensors (Figure 4 and Figure 5) with the as prepared nanoparticle films.

**Figure 6:**
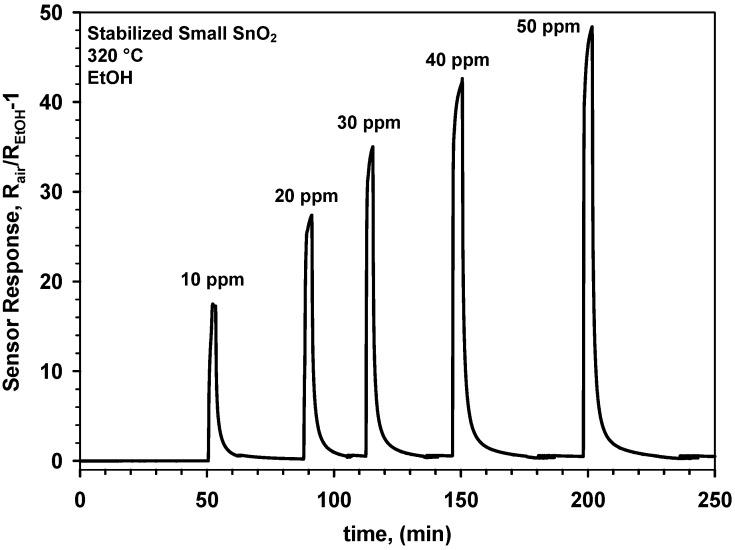
Response of a gas sensor made of small SnO_2_ nanocrystals to ethanol after a 12 h sintering step at 600 °C.

Figure 6 shows the response to increasing EtOH concentrations of a SnO_2_ sensor made of small nanoparticles that was sintered at 600 °C for 12 h prior to gas sensing. The sensor shows (Figure 6) similar response and recovery time to that having an as prepared SnO_2_ nanoparticle film (Figure 5). This is in agreement with the identical surface composition and film thickness of the as prepared and sintered SnO_2_ films. However, the magnitude of the sensor response was drastically increased with respect to the latter. In particular, the sintered sensor (Figure 6) had a response of nearly 17 to 10 ppm of EtOH while the as prepared barely reached 8. This is surprising as high temperature sintering is expected to increase crystal size leading to lower sensitivity [3,5]. Here, it is suggested that the enhancement of the sensor response arise from the formation of sintering necks with size below that of the main grains between the SnO_2_ particles. Formation of partially or fully depleted sintering necks can increase the sensitivity of metal-oxide gas sensors and can hardly be measured by XRD or nitrogen adsorption [7]. As a result, two instability mechanisms are suggested for the pure SnO_2_ nanoparticles. A first, where the average crystal (and grain) size is increased (Figure 4) resulting in a drop of the sensor response, and a second, where partially depleted sintering necks are formed increasing the sensitivity. Similar effects were observed for size selected SnO_2_ agglomerates [21]. There, very small changes in the sintering properties of the agglomerates that could hardly be tracked by XRD analysis led to drastic variations in their sensing response to EtOH. Both dynamics can be accelerated by a pre-sintering step leading to (Figure 6) higher response and more stable sensing properties.

**Figure 7:**
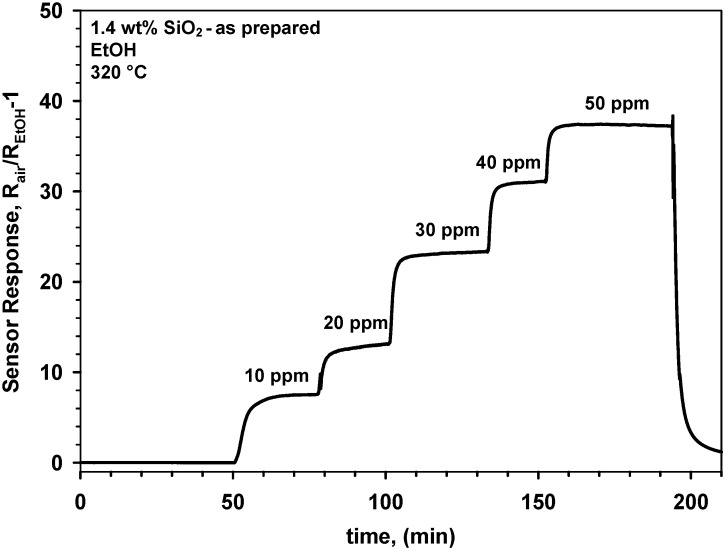
Response of a gas sensor made of as prepared 1.4 wt% SnO_2_-SiO_2_ nanocomposites to increasing EtOH concentrations in dry air.

The sensing dynamics of the SnO_2_-SiO_2_ composites was different from that of the pure SnO_2_ nanoparticles (Figure 4, Figure 5 and Figure 6). To better investigate the effect of SiO_2_ cosynthesis on the sensing properties, all synthesis parameters were kept constant and only the Si-content was varied. Figure 7 shows the response to step-wise increases in EtOH concentration of a sensor made of as prepared 1.4 wt% SnO_2_-SiO_2_. The response and recovery times of these nanocomposites (Figure 7) were comparable to that of the pure SnO_2_ (Figure 5). Nevertheless, the magnitude of the sensor response was initially lower than that of the SnO_2_ nanoparticles (Figure 5) reaching about 37 at 50 ppm EtOH (Figure 7). This is in contrast to the smaller crystal size of these nanocomposites (Figure 3, triangles up) that should lead to higher sensitivity [5]. This is attributed to the sintering inhibition effect of the SiO_2_ that may have limited the growth of sintering neck between the main SnO_2_ grains.

**Figure 8:**
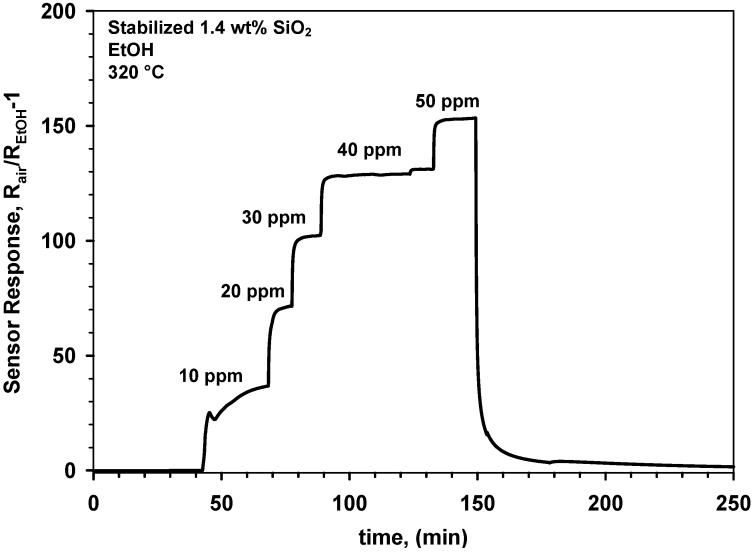
Response to increasing EtOH concentrations in dry air of a gas sensor made of 1.4 wt% SnO_2_-SiO_2_ nanocomposites upon stabilization by sintering for 12 h at 600 °C.

After sintering these films at 600 °C for 12 h, their response was greatly increased (Figure 8). More in details, upon this stabilization step, the response of the 1.4 wt% SnO_2_-SiO_2_ to 50 ppm EtOH increased from 37 (Figure 7) to 153 (Figure 8). This 4 fold increase in sensitivity is in line with the reported optimal Si-doping of SnO_2_ nanoparticles [7]. Furthermore, it confirms the long-term instability mechanisms observed for pure SnO_2_. As the presence of SiO_2_ drastically inhibit the crystal growth, the sintering necks formed at 600 °C are smaller and thus more depleted than for pure SnO_2_(Figure 5 and Figure 6) resulting in a more drastic enhancement of their sensing performance (Figure 7 and Figure 8). A more detailed analysis of the neck morphologies and growth dynamics is required to quantitatively describe the sensing response enhancement of these nanocomposites [4]. Higher SiO_2_ contents (Figure 3), up to 4 wt%, resulted in a similar enhancement of the sensing properties. Overall, cosynthesis of SiO_2_ increases the variation of sensor resistance during injection of EtOH concentration as it was previously investigated in details [7].

### 3.3. Cross-Sensitivity to Humidity

**Figure 9:**
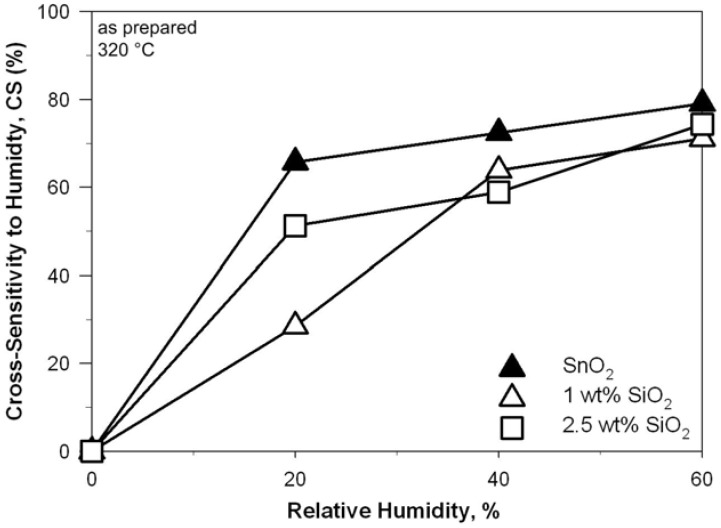
Cross-sensitivity to relative humidity (20 °C) of as prepared SnO_2_ nanoparticles (solid triangles), and of 1 wt% (empty triangles) and 2.5 wt% (squares) SnO_2_-SiO_2_ nanocomposites at 320 °C.

The stability of the sensor response toward variations in relative humidity is of major importance for several applications [2,22]. Doping of SnO_2_ nanoparticles with Ti has been reported to drastically decrease their cross-sensitivity to humidity [9]. Recently, this has been attributed to thermodynamically dictated enrichment of the SnO_2_ surface with Ti atoms [23] that lower the adsorption energy of dissociatively adsorbed H_2_O [24]. The effect of SiO_2_ cosynthesis on the cross-sensitivity (CS) to humidity of SnO_2_ nanoparticles, however, has not been investigated yet. This is particularly important as flame-made SiO_2_ has high surface concentration of hydroxyl groups that facilitate the binding of H_2_O molecules [10] and thus may result in a strong enhancement of the CS to humidity. Figure 9 shows the CS to humidity during EtOH detection, defined as change of the sensor response in dry air (Equation (2)), of the pure SnO_2_ (triangles solid), 1 wt% (empty triangles) and 2.5 wt% (empty squares) SnO_2_-SiO_2_ nanocomposites as a function of the relative humidity. The CS to humidity of the pure SnO_2_ sensors (Figure 9, solid triangles) increased from 51 to 74% with increasing r.h. from 20 to 60%. This is in agreement with the drastic change in sensor response reported for SnO_2_ nanoparticles with increasing r.h. content [9]. The continuous increase in CS above 20% r.h. (Figure 9, solid triangles) indicates that the SnO_2_ surface has not yet been saturated with adsorbed H_2_O species. More important, the CS of both SnO_2_-SiO_2_ sensors (Figure 9, empty squares and triangles) was comparable to that of the pure SnO_2_ (Figure 9, solid triangles). This is different than the effect of Ti-doping [7] and in contrast to the super-hydrophilic properties of flame-made SiO_2_ [10]. However, as SiO_2_ is an isolator, localized SiO_2_ molecules/clusters on the SnO_2_ surface may act as active sites for H_2_O binding but still have minimal impact on the sensing properties of the SnO_2_ nanocrystals due to its inefficient electron conduction properties. As a result, SiO_2_ cosynthesis leads to the same CS than pure SnO_2_ nanoparticles. This is in contrast to the modification of SnO_2_ crystals with hydrophilic zeolites [25,26] where notable variations from the sensing response of the pure SnO_2 _were observed. Minimization of the CS while improving the long-term stability and sensitivity of SnO_2_-based gas sensors may be achieved by synthesis of Sn_1__−x_Ti_x_O_2_-SiO_2_ nanocomposites [9]. 

It should be pointed out that the sensing chamber geometry has considerable influence on the resulting sensor response dynamics as previously discussed in details [27]. The setup utilized here was previously tested for several other materials [7,9,15,17] and, for the utilized total gas flow (1 L/min), results in reaction limited sensor responses [27].

## 4. Conclusions

The long-term structural stability of small SnO_2_ nanoparticles has been investigated for temperatures relevant to metal-oxide gas sensor operation. A stable SnO_2_ crystal size of *ca*. 24 nm has been determined for operation at up to 400 °C. This stable size was decreased to *ca*. 11 nm by cosynthesis of 1.4 wt% SiO_2_. However, the slow asymptotic-like growth of the SnO_2_ crystals indicated poor long-term stability even for such thermodynamically more stable nanocomposites. This was further confirmed by investigation of the sensing response of the as prepared SnO_2_ nanoparticle sensors. Two main instability mechanisms were suggested: first a response drop due to crystal growth and then a response enhancement due to the formation of full or partially depleted sintering necks. Analysis of the performance of SnO_2_-SiO_2_ nanocomposites confirmed that sintering the films at 600 °C for 12 h prior gas sensing increases sensor stability and performance. Furthermore, the effect of SiO_2_ cosynthesis on the cross-sensitivity to humidity of SnO_2_-based gas sensors was investigated. Cosynthesis of up to 2.5 wt% SiO_2_ had no effect on the sensor cross-sensitivity to humidity suggesting minimal electronic interaction between SnO_2_ and SiO_2_. These result shows that SnO_2_-SiO_2_ nanocomposites can enhance the long-term stability and sensitivity of SnO_2_-based gas sensors while having minimal impact on the residual SnO_2_ properties. 
